# Giant mucinous cystadenoma: a case report

**DOI:** 10.1186/s13256-019-2102-z

**Published:** 2019-06-14

**Authors:** Chipo Gwanzura, Annie Fungai Muyotcha, Thulani Magwali, Zvavahera Mike Chirenje, Mugove Gerald Madziyire

**Affiliations:** Department of Obstetrics and Gynecology, College of Health Sciences, University of Zimbabwe, P.O. Box A178, Avondale, Harare Zimbabwe

**Keywords:** Giant mucinous cystadenoma, Resource-limited, Health-seeking behavior

## Abstract

**Introduction:**

Giant ovarian cysts are rarely described in the literature, owing to the availability of advanced imaging technologies in developed countries leading to early treatment. In resource-limited settings, various factors lead to late presentation.

**Case presentation:**

We present a case of a 48-year-old black African woman with a giant mucinous cystadenoma who presented to a tertiary hospital with massive abdominal distention 5 years after being referred from a district hospital for the same problem. Surgical management resulted in fatal complications.

**Conclusions:**

The surgical management of these huge tumors is associated with many life-threatening complications. Transvaginal ultrasound should be used in resource-limited settings to delineate ovarian masses. Community health workers must be involved in scouting and follow up of community members with unusual abdominal swellings in developing countries to avoid delays in care.

## Introduction

Tumors of the ovary presenting with diameters greater than 10 cm are referred to as giant ovarian cysts [1]. These are rarely seen in high-income countries and consequently are rarely described in the literature, owing to availability of resources and advanced imaging technologies leading to early diagnosis of small or medium-sized tumors [2].

These tumors are generally asymptomatic at early stages, causing symptoms only after reaching enormous dimensions, and consequently are diagnosed late in low- and middle-income countries (LMICs) [1]. Compressive symptoms or a visible abdominal mass are the most frequent presenting complaints [2].

The surgical management of these masses is associated with many life-threatening complications, which arise predominantly after surgery owing to rapid changes in body circulation, and with pulmonary edema. The former include severe hypotension, increased venous return, cardiac failure, respiratory failure, and intestinal distention [2].

We report a case of a 48-year-old woman with a giant mucinous cystadenoma who presented with massive abdominal distention and whose surgical management resulted in fatal complications. We aim to remind clinicians of the challenges in diagnosing, managing, and precautions to be taken when performing surgery for patients with giant ovarian cysts.

## Case presentation

A 48-year-old, para 5, postmenopausal black African woman who was human immunodeficiency virus (HIV)-negative presented to our casualty department with a 5-year history of progressive abdominal swelling. Five years prior to presenting, which was 1 year before she reached menopause, she had noticed that her abdomen was gradually distending. Her symptoms were associated with constipation, feeling of incomplete rectal emptying, early satiety, vomiting, and urinary frequency and urgency. She did not have any chronic illnesses and had a negative personal and family history of ovarian, uterine, bowel, and breast cancers. She was not receiving any medication prior to this presentation. She had delivered five children by cesarean section, and they were all alive and well. She lived in a rural area and was a subsistence farmer. She did not smoke and did not drink alcohol.

She had ascitic taps three times in 1 week at a district hospital before referral to a higher-level hospital because of recurrent reaccumulation of ascites. A transabdominal ultrasound scan (USS) showed generalized ascites with a thick fluid with septa, as well as bilateral mild hydroureter and hydronephrosis. Again, the ascites was drained twice. One month later, she underwent computed tomography (CT), which showed a large predominantly cystic lesion that occupied almost the entire abdominal and pelvic cavities, which were distended, causing a marked mass effect on surrounding organs and bowel. The lesion had areas of internal septation predominantly on the right flank with no features of metastatic disease. Tumor markers measured during this admission are shown in Table 1.

**Table 1:** 2013 Tumour marker results

Tumor marker	Result	Normal range
Lactate dehydrogenase (LDH)	477 U/L	120–250 U/L high
α-Fetoprotein (αFP)	2.31 ng/ml	0–6 ng/ml
Serum β-human chorionic gonadotropin	0.28 mIU/ml	< 5 mIU/ml
Carcinoembryonic antigen (CEA)	115.9 U/ml	0–37 U/ml high
Cancer antigen 125 (CA 125)	143.9 U/ml	0–35 U/ml High

**Fig. 1:**
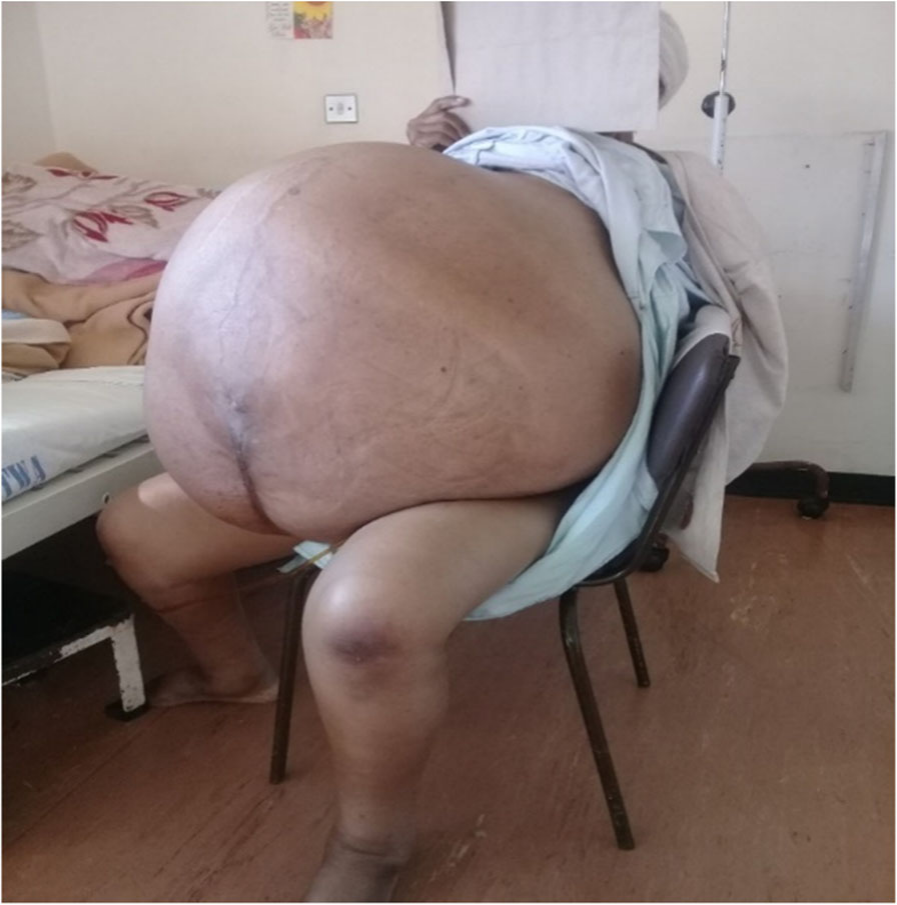
Grossly distended abdomen before surgery

The patient was referred to a tertiary hospital but only went 5 years later. Upon admission, she had marked temporal wasting, with bilateral pitting lower limb edema extending to her sacrum. She had a normal breast examination. Her blood pressure was elevated at 167/93 mmHg, with tachycardia of 150 beats/min. Her body temperature was 36.8 °C. She had equal air entry bilaterally, and her cardiorespiratory and neurological systems were normal. She had a lower midline scar with massive abdominal distention that was nontender and had a positive fluid thrill test result (Fig. 1).

Results of investigations showed microcytic anemia with hemoglobin of 9.9 g/dl, white blood cell count of 7 × 10^3^/μl, and platelet count of 250 × 10^3^/μl. She had normal urea and electrolytes and hypoalbuminemia of 21 g/L, with results of the rest of her liver function tests being normal. Urinalysis did not show abnormalities.

Her case was discussed by a multidisciplinary team (MDT) that included gynecological oncologists, radiation oncologists, general surgeons, anesthetists, and nursing staff. The MDT considered the risks of death following hemodynamic instability and bleeding as well as the postoperative risks of deep vein thrombosis, pulmonary embolism, difficulty weaning off the ventilator, and death. The conclusion of the meeting was to take the patient for staging laparotomy despite these risks.

Preoperatively, the patient and her relatives were counseled on the possible complications. The patient received a transfusion with 2 Units (U) of packed cells (PCs). The team also contemplated drainage of the mass preoperatively but was unable to secure appropriate drains to drain the thick green fluid from the mass.

**Fig. 2:**
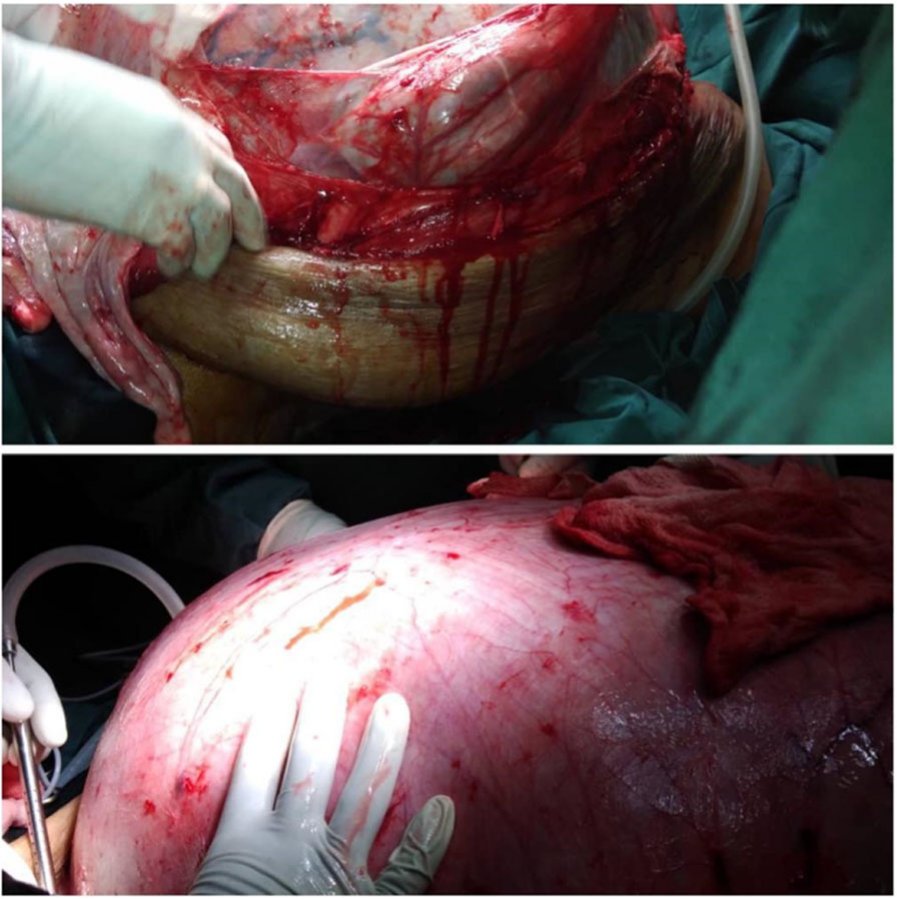
Intraoperative findings upon abdominal entry

At laparotomy, through a right paramedian incision, a huge abdominopelvic mass was found filling the whole abdomen and pelvis. The liver, spleen, and hemidiaphragms looked normal. The mass was shelled out by blunt dissection (Fig. 2). The mass burst during mobilization, however, with rapid drainage of dark-colored fluid and decompression 50 min into the surgery. Subsequently, the mass was stripped off the anterior abdominal wall and completely excised. A total abdominal hysterectomy was done. The anterior abdominal wall was noted to have very deficient layers. Estimated blood loss was 450 ml. Anesthetically, the patient was unstable, particularly after the rapid decompression. She received a massive transfusion of 7 U of PCs, 6 U of fresh frozen plasma, and 4 L of gelafundin with 7 L of Ringer’s lactate. The surgery lasted about 3 hr. The patient was admitted to the intensive care unit (ICU) for cardiopulmonary support.

**Fig. 3:**
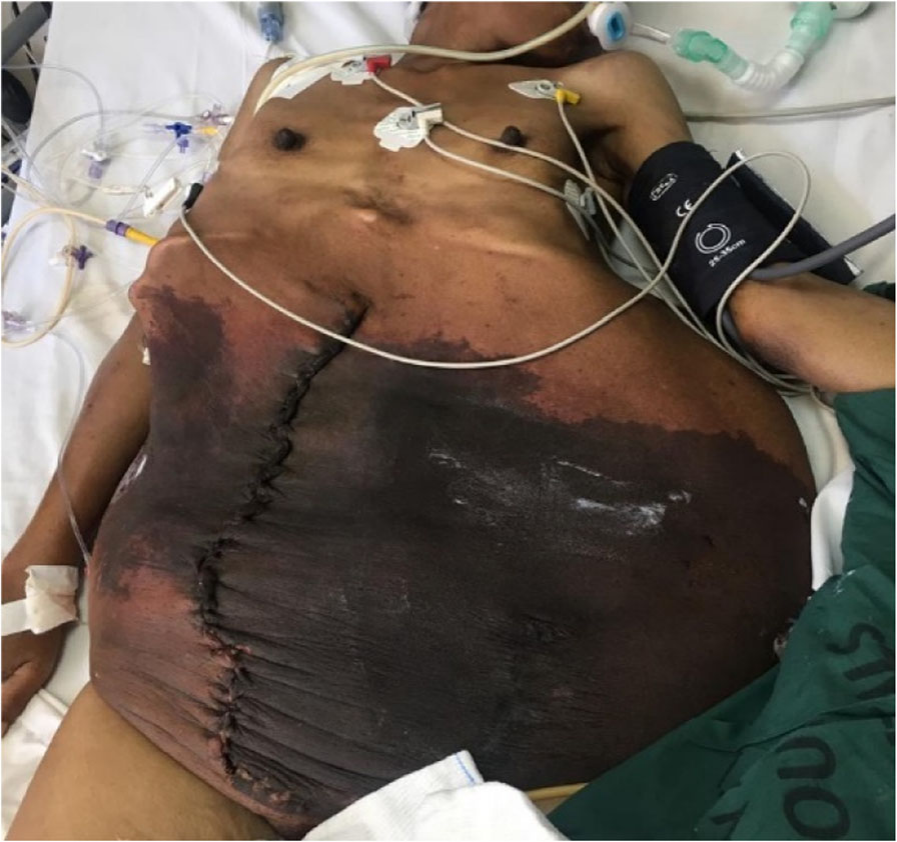
Gangrenous abdominal wall postsurgery

The patient was critically ill in the ICU. On the day of admission, she had a cardiac arrest and was successfully resuscitated. She required inotropic support and ventilation. By day 5, she had developed hepatosplenomegaly, uremia, and an increased international normalized ratio. She was leaking serous fluid from the suture line, with darkening of the previously stretched skin of the anterior abdominal wall. She remained anemic with thrombocytopenia. She received a further 6 U of PCs. She was kept on albumin and given high-protein energy feeds by the dietitian in view of her hypoalbuminemia and malnutrition. By day 6, she was noted to have disseminated intravascular coagulopathy. She was given vitamin K. A pneumothorax was noted, and the cardiothoracic surgeon inserted a chest drain. She was also noted to have aspiration pneumonia following a self-extubation on the same day. By day 10, she was noted to have multiple organ failure with oliguria, falling level of consciousness, aspiration pneumonia, a gangrenous abdominal wall (Fig. 3), and coagulopathy.

**Fig. 4:**
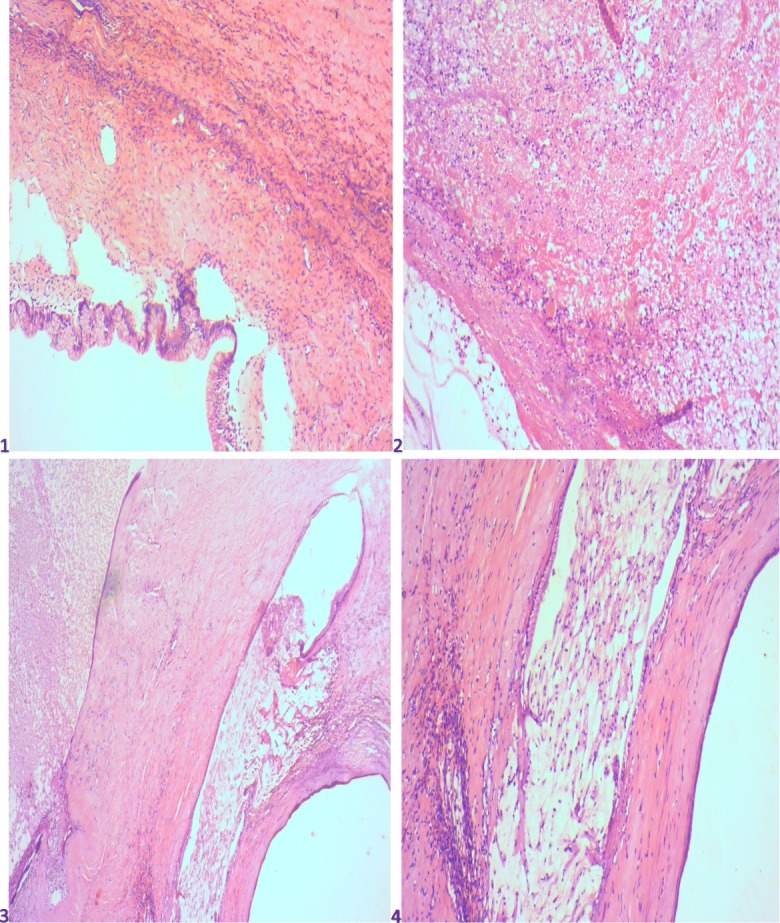
Histological plates 1–4 showing partial cysts lined by mucus-secreting columnar cells with no stromal invasion, stratification, or atypia

**Fig. 5:**
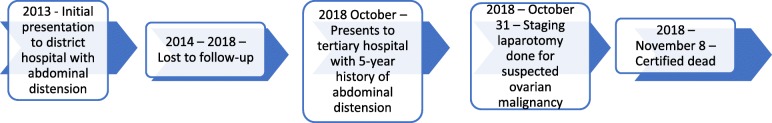
Timeline showing sequence of events

She required inotropic support and ventilation. She died on day 10 after surgery. The pathology report showed a partial cystic lesion with benign epithelia of mucin-secreting columnar cells, no evidence of stromal invasion, no stratification, and no atypia and intraluminal mucin (Fig. 4). The cyst walls were necrotic with fibrinopurulent exudate. Normal ectocervical and endocervical mucosa was observed. The endometrium was inactive, and there was evidence of an anterior fibroid. This confirmed the diagnosis of benign mucinous cystadenoma. Figure 5 illustrates the sequence of events.

## Discussion

We report a case of a 48-year-old woman with a giant mucinous cystadenoma who presented with massive abdominal distention and whose surgical management resulted in fatal complications. Benign mucinous cystadenomas make up the majority (81%) of mucinous tumors. These tumors can grow to extremely large sizes and are among the largest of any recorded tumor in the body [3], as was the case with our patient. They usually occur as large multicystic masses with mucus-containing fluid, presenting commonly in women in their 20s to 40s. The large size can itself be suggestive of mucinous histology. Our patient’s presentation was pathognomic: massive abdominal distention caused by the huge mass with associated symptoms of compression of adjacent organs, constipation and rectal tenesmus from colonic compression, early satiety, and vomiting from gastric compression, frequency and urgency from increased pressure on the bladder, ureteral obstruction with the hydroureter seen on a USS, and lower limb edema from pressure on the draining veins and lymphatic vessels [4, 5].

This patient presented to the tertiary hospital 5 years after being referred from the district hospital. In low-resource settings, late presentation is a common feature due to socioeconomic factors as well as cultural beliefs and fear of surgery leading most to present when the symptoms become unbearable [1]. It is therefore imperative that community health workers be involved in scouting and follow-up of community members with unusual abdominal swellings.

Among the tumor markers measured in our patient, lactate dehydrogenase (LDH), carcinoembryonic antigen (CEA), and cancer antigen 125 (CA 125) were elevated. At the time the tests were done, the patient was 43 years old and premenopausal. The CA 125 assay is unreliable in differentiating benign from malignant ovarian masses in premenopausal women because of the increased rate of false-positive results and reduced specificity [6]. When CA 125 is less than 200 U/ml as in our patient, further investigations are appropriate. LDH, β-human chorionic gonadotrophin, and α-fetoprotein should be measured in all women under age 40 with a complex ovarian mass because of the possibility of germ cell tumors [7]. In postmenopausal women, CA 125 should be used for primary evaluation because it allows the Risk of Malignancy Index of ovarian cysts to be calculated [8]. However, CA 125 should not be used in isolation, because it is nonspecific for ovarian cancer. There is not enough evidence to suggest that panels including multiple tumor markers will offer any further advantage in the initial assessment of ovarian cysts in postmenopausal women, because all the markers show low sensitivity and wide variation in specificity when used in isolation or in combination with CA 125; hence, their routine use is not recommended [9]. The elevated CEA in our patient supported the diagnosis because it is more likely to be elevated in 88% of cases vs 19% of nonmucinous ovarian carcinomas [5].

Our patient’s case displays the shortfalls of transabdominal USS as an imaging modality for the assessment of suspected ovarian masses. Although a pelvic ultrasound is the single most effective way of evaluating an ovarian mass, transvaginal ultrasonography is preferable because of its increased sensitivity over transabdominal ultrasound. A transabdominal USS should not be used in isolation; it should be used to provide supplementary information to transvaginal ultrasound, particularly when the ovarian cyst is large [10]. Our patient’s case shows the utility of a CT scan if malignant disease is suspected, because it was able to define the large cystic mass.

As in our patient’s case, most mucinous tumors (about 79%) are unilateral. An algorithm was proposed in which a tumor greater than 10 cm correctly predicted primary ovarian origin in 82% of cases [4].

Our team considered aspiration of the tumor prior to surgery. However, some authors state that aspiration of abdominal cysts should be avoided because this could cause complications such as infections, bleeding, cyst rupture, increased peritoneal adhesions, or possible dissemination of malignant cells. They therefore consider preoperative and intraoperative drainage unsafe [1]. In the past, it was thought that pseudomyxoma peritonei resulted from mucinous ovarian tumors, but now it is believed to be derived universally from appendicular low-grade (adenomatous) mucinous tumors; the ovarian involvement is secondary [3]. Other surgeons advocate controlled fluid aspiration intraoperatively to reduce hypotension and low caval reload related to sudden drop of abdominal pressure. Draining the mass is an extreme option for selected unresectable cases with high wall tension [2].

The gold standard of treatment of any suspected ovarian mass includes intact removal of the involved adnexa with intraoperative pathological evaluation, typically laparotomy, total hysterectomy, bilateral salpingo-oophorectomy, and staging procedure, including lymphadenectomy [4]. Surgery in patients with such huge masses has a high risk, as evidenced by the complications experienced by our patient. Fatal complications described in the literature include pulmonary and cardiac failure, pulmonary embolism, and sepsis. In one case with good prognosis, part of the patient preparation prior to surgery included lung preparation to improve pulmonary function postsurgery [2]. This may involve incentive spirometry to breathe deeply and exercise the lungs before and after surgery [11, 12].

Adequate supportive care while resecting giant ovarian masses is of the utmost importance. Postoperatively, reducing ileus, providing respiratory support, supporting abdominal wall tension, and monitoring hemodynamic parameters are key [2]. After tumor excision, an abdominal wall reconstruction might be necessary because of the laxity and redundancy of the skin [13]. We planned reconstructive surgery of the anterior abdominal wall after initial postoperative recovery because the patient was unstable at primary surgery.

When the abdomen is entered surgically, care should be taken to remove the ovary intact without spillage of the cyst contents, because rupture of a stage I mucinous ovarian carcinoma may increase its potential for recurrence. Benign mucinous cystadenomas are by definition confined to the ovary, and no further procedure is required. Therefore, had a frozen section been available, this diagnosis would possibly have enabled an oophorectomy to be performed, and the hysterectomy that contributed to additional theater time could have been avoided. Consideration also needs to be given to the fact that though benign, borderline to invasive disease may exist as a continuum in a cystadenoma. Only a limited number of sections can be evaluated intraoperatively because these tumors are quite large; hence, this may contribute to inaccuracies in diagnosis with frozen sections. Although appendectomy was previously performed for any ovarian tumor with mucinous histology, including benign lesions, current data do not support the practice [4].

The mass was not weighed, because it ruptured and was drained intraoperatively. A review of 20 cases with lesions exceeding 20 kg confirmed a low malignancy rate [2]. Other authors also concur, stating that huge intra-abdominal tumors that almost double a patient’s body weight can hardly be malignant [13].

## Conclusion

Benign mucinous cystadenomas can grow to massive sizes, particularly in LMICs, and provide a huge surgical management challenge. Transvaginal ultrasound should be employed early to clearly delineate the ovaries, particularly in resource-limited nations. Community health workers in developing countries must be involved in scouting and follow-up of community members with unusual abdominal swellings to avoid delays in care.

## Abbreviations

CA 125: Cancer antigen 125
CEA: Carcinoembryonic antigen
CT: Computed tomography
HIV: Human immunodeficiency virus
ICU: Intensive care unit
LDH: Lactate dehydrogenase
LMICs: Low- and middle-income countries
MDT: Multidisciplinary team
PCs: Packed cells
USS: Ultrasound scan
